# Properties of Fe–Si Alloy Anode for Lithium-Ion Battery Synthesized Using Mechanical Milling

**DOI:** 10.3390/ma15051873

**Published:** 2022-03-02

**Authors:** Kikang Lee, Jejun Jeong, Yeoneyi Chu, Jongbeom Kim, Kyuhwan Oh, Jeongtak Moon

**Affiliations:** 1 Research and Development Center, MK Electron, Yongin 17030, Korea; kikang86@snu.ac.kr (K.L.); yychu@mke.co.kr (Y.C.); 2 Department of Materials Science and Engineering, Seoul National University, Seoul 08826, Korea; triplej@snu.ac.kr (J.J.); triplucky@snu.ac.kr (J.K.); kyuhwan@snu.ac.kr (K.O.)

**Keywords:** anode material, lithium-ion battery, mechanical milling, silicon nanocomposite, iron silicide

## Abstract

Silicon (Si)-based anode materials can increase the energy density of lithium (Li)-ion batteries owing to the high weight and volume capacity of Si. However, their electrochemical properties rapidly deteriorate due to large volume changes in the electrode resulting from repeated charging and discharging. In this study, we manufactured structurally stable Fe–Si alloy powders by performing high-energy milling for up to 24 h through the reduction of the Si phase size and the formation of the α-FeSi_2_ phase. The cause behind the deterioration of the electrochemical properties of the Fe–Si alloy powder produced by over-milling (milling for an increased time) was investigated. The 12 h milled Fe–Si alloy powder showed the best electrochemical properties. Through the microstructural analysis of the Fe–Si alloy powders after the evaluation of half/full coin cells, powder resistance tests, and charge/discharge cycles, it was found that this was due to the low electrical conductivity and durability of β-FeSi_2_. The findings provide insight into the possible improvements in battery performance through the commercialization of Fe–Si alloy powders produced by over-milling in a mechanical alloying process.

## 1. Introduction

The use of secondary batteries is emerging as a critical national strategic industry because of the universalization of mobile devices and the need to save energy and protect the environment. The compact mobile device market is dominated by lithium (Li)-ion batteries, and with continuous improvements in the performances of these batteries, the medium-to-large battery market for electric and hybrid electric vehicles is expected to grow [1,2,3,4].

Li-ion batteries are composed of an anode, cathode, electrolyte, and separator, and carbon materials, such as graphite, are mainly used in the cathode. Carbon-based anode materials have many advantages besides their low price and high stability. However, because their theoretical capacity is limited to 372 mAh/g, their use is limited in current applications that require high capacity [5,6,7]. Therefore, many studies have been conducted to find materials that can replace carbon-based anode materials.

The representative alloy elements Si, Ge, and Sn show excellent characteristics in terms of having a high capacity per unit weight and volume compared to existing graphite anode materials. Therefore, they are drawing attention and actively being researched for use as materials in next-generation high-capacity Li-ion secondary batteries (Si > 4000, Ge > 1500, Sn > 900 mAh/g) [1,8,9].

As an anode material, Si shows a higher capacity per unit weight and volume than graphite because it forms an Li22Si5 alloy by accepting up to 4.4 Li atoms. Moreover, since it is known to have the lowest discharge voltage, it is the most appropriate anode material for Li secondary batteries that require high energy density [10,11,12,13].

Despite having high-capacity characteristics, it is difficult to use Si commercially because a large structural volume expansion occurs when it is alloyed with Li. When a Li metal is formed, the active anode material not only accumulates Li ions but also accepts the same number of electrons that the Li ions do. In other words, the active anode material transitions to an anionic state with a larger radius than that of a neutral atom because of the movement of the electric charge. As a result, the volume of the metal with Li ions increases by 150–400% compared to that of the metal without Li ions (the volume expansion rate is 400% for Si). Such an increase in volume causes cracks and mechanical failure in the material. The surface of the new Si generated by mechanical failure comes in contact with the electrolyte in the battery to generate a solid electrolyte interface (SEI) layer. This SEI layer has insulating properties and depletes the electrolyte, resulting in a rapid decrease in capacity within the charge and discharge cycles [1,14,15,16,17,18].

For this reason, active/inactive Fe–Si alloys that have been produced in various ways for their advantageous properties, such as high electronic conductivity that can significantly improve the electrochemical performance of Si-based materials, have been studied in the past few decades [19,20,21,22]. Among these processes, the mechanical alloying process can form an alloy with a high melting point compound by repeating the process of combining different types of metal powders and crushing them using the mechanical impact from the ball mill. In addition, it is a very efficient and productive process in that it is carried out at room temperature [23,24,25].

Most studies have focused on sturdy iron silicide formation and decreasing the Si phase size (nanoscale) by increasing the mechanical alloy milling time to improve battery performance [14,26,27]. However, we believe that concentrating on the formation of various essential new phases as the mechanical alloy milling time increases can contribute to more diversified and improved research on anode materials.

In this study, the active Si content was reduced using high-energy machine milling, and Fe–Si alloy powders were designed wherein Si nanoparticles were finely dispersed in an inactive (less active) matrix (iron silicide) to stabilize the electrochemical cycling performance. Fe–Si alloy powder was produced, wherein Si was nano-crystallized using a mechanical alloying process, and a matrix (iron silicide) was formed through its reaction with Fe. When Si reacts with Li, it is designed such that the matrix can suppress minimized volume expansion [26,28,29,30,31]. Afterwards, comprehensive studies were conducted to evaluate how the electrochemical performance was affected by several factors, such as milling time (milling energy), the grain size of the Si phase, and new matrix (silicide phase) generation due to over-milling [20,27,32,33].

## 2. Materials and Methods

### 2.1. Synthesis of Fe–Si Nanocomposite Materials

The raw material alloys were supplied by MK Electron (Korea), a manufacturer of Si-based anode active materials. The Fe–Si alloy was melted via the vacuum induction melting process at a ratio of 85 at.% Si and 15 at.% Fe. The alloys used in the mechanical synthesis process became 1 mm or smaller alloyed powders through crushing and grinding after solidification. The mechanical alloy method was used to produce the Si alloy anode material of the powders by performing milling after charging the powders, balls (media), and a process control agent (PCA). The PCA played the role of preventing the agglomeration and solidification of the powders due to excessive pressure welding during milling [25].

Mechanical synthesis was performed via a high-energy milling process using a Simoloyer CM20 (Zoz GmbH, Wenden, Germany). The balls and powder were charged at a ball-to-powder ratio of 15:1. To carry out the role of the PCA, stearic acid (Samchun Chemical, Seoul, Korea) was added to 3.6 wt.% of the charged powder. After charging the alloy powders, balls, and PCA into the milling equipment, they were rotated at 650 rpm using a rotor. Then, the Fe–Si alloy powders were collected at different milling times for up to 24 h.

**Figure 1:**
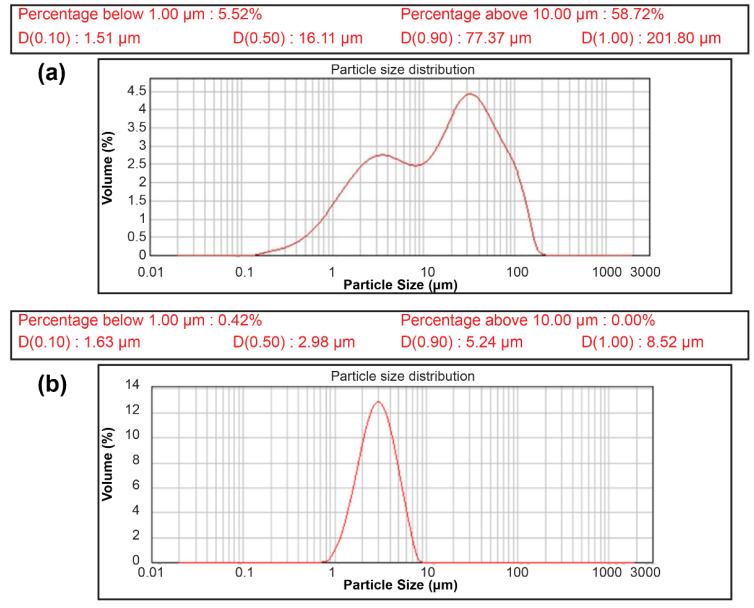
Particle size distribution curve of the Fe–Si alloy powders manufactured through the mechanical synthesis process. Particle size distribution graphs immediately after (**a**) mechanical milling and (**b**) particle size control.

The powders collected in this manner exhibited irregular particle sizes. The particle size was controlled for all the samples prepared for different time periods because it could affect the evaluation of the resulting electrochemical properties. The Fe–Si alloy powders with non-uniform particle sizes and coarse distribution in Figure 1a were ground into the final versions with a uniform particle size distribution and classified using the Air Jet Mill (STJ-200, JS Tech, Chungcheongbuk-do, Korea), as shown in Figure 1b. The Air JET Mill equipment operating experimental conditions had a total of three factors: (1) powder feed speed, (2) air flow forming air press (pusher press), and (3) grinding air press. The conditions of the air jet mill for manufacturing the material of this study are: (1) raw material input rate: 1 kg/h, (2) push press: 0.7 mPa, and (3) grind press: 0.4 mPa.

### 2.2. Material Characterization

X-ray diffraction analysis (D8, Bruker AXS, Karlsruhe, Germany) was performed using Cu-Kα radiation with a wavelength of 0.15418 nm to analyze the phase and crystallinity of the Fe–Si alloy powders manufactured by the mechanical alloy process. The powder pattern was measured in the 2θ range of 10–60° with a measurement time of 2.5 s per step at a step size of 0.045°.

The morphology of the manufactured Fe–Si alloy powders was analyzed using a field-emission scanning electron microscope (JSM-6701F, JEOL, Tokyo, Japan) and a field-emission gun transmission electron microscope (JEM-3000F, JEOL, Tokyo, Japan). 

The resistance of the Fe–Si alloy powders was measured (RS8-1G, DSA, Chungcheongbuk-do, Korea) using the 4-terminal sensing method. To make the powder interface as close as possible, up to 2 g of powder was put into a mold, and 408 kg/cm^2^ of pressure was applied to produce samples in the form of pellets, and then their resistance was measured.

### 2.3. Electrode Preparation

Two types of anode electrodes were fabricated to evaluate the electrochemical properties of the Fe–Si compounds manufactured in this study. To evaluate the intrinsic initial capacity and initial efficiency and analyze the deteriorated cross-sections of the particles after sufficient cycles, electrodes with 80 wt.% Fe–Si alloy powders were fabricated according to the method described in Section 2.3.1. In addition, to evaluate the cycling performance of the particles, electrodes with 15 wt.% Fe–Si alloy powders were fabricated according to the method described in Section 2.3.2. 

#### 2.3.1. Electrode Preparation of 80 wt.% Fe–Si

For electrochemical evaluation, 80 wt.% of active material (Fe–Si compound produced by mechanical synthesis), 15 wt.% of polyacrylic acid binder (PAA, AST-9005, AEKYUNG CHEMICAL, Seoul, Korea), and 5 wt.% of conductive agent (Super P, TIMCAL, Tokyo, Japan) were prepared. First, the PAA binder and conductive agent were mixed at a rate of 2000 rpm for 5 min using the Thinky Mixer (ARE-310, THINKY, Laguna Hills, CA, USA), then the active material was added and mixed at 2000 rpm for 5 min. The mixture was mixed at 2000 rpm for 10 min after adding distilled water to obtain a solid content of approximately ~30 wt.%. It was then cast on a smooth Cu foil (18 μm-thick, UACJ, Tokyo, Japan) using the doctor-blade method. An electrode was fabricated by adjusting the blade gap such that the mixed slurry would become 3.5 mg/cm^2^. The electrode was then dried at 110 °C in air atmosphere for 10 min and then punched. The electrode that was punched in a circle with a diameter of 16 mm was rolled to 1.5 g/cm^3^, and then spot-welded in a CR2032 coin cell case. It was then dried at 110 °C under vacuum for 12 h.

#### 2.3.2. Electrode Preparation of 15 wt.% Fe–Si

For the evaluation of life characteristics, 15 wt.% of active material (Fe–Si alloy powder produced by mechanical synthesis), 81 wt.% of graphite (95 wt.% of 918 (II), BTR, Shenzhen, China and 5 wt.% of SFG6, TIMCAL, Tokyo, Japan), 1.5 wt.% of CMC (350HC, Nippon paper, Tokyo, Japan), and 1 wt.% of conductive agent (Super P Li, TIMCAL) were mixed at 2000 rpm for 3 min using the Thinky Mixer (ARE-310, THINKY, Laguna Hills, CA, USA). Distilled water was then added such that the solid content was ~60 wt.%, and the mixture was mixed at 2000 rpm for 3 min. Lastly, 1.5 wt.% SBR (BM400-B, Zeon, Tokyo, Japan) was added, and the mixture was mixed at 2000 rpm for 3 min. It was then cast on a smooth Cu-foil (10 μm-thick, UACJ, Tokyo, Japan) using the doctor-blade method. An electrode was fabricated by adjusting the blade gap such that the mixed slurry would be 7 mg/cm^2^. The electrode was then dried at 110 °C in atmosphere for 10 min and then punched. The electrode that was punched in a circle with a diameter of 16 mm was rolled to 1.6 g/cm^3^, and then spot-welded in a CR2032 coin cell case. It was then dried at 110 °C under vacuum for 12 h.

### 2.4. Cell Assembly

Every electrochemical evaluation was conducted using a CR2032 coin cell case. The cells were assembled inside a glove box (KK-021-AS, Koreakiyon, Seoul, Korea) filled with argon, and the water and oxygen contents were maintained below 1 ppm. First, a half-cell was fabricated to evaluate the initial capacity and efficiency using a 0.45 mm-thick Li metal chip (EQ-Lib-LiC45, MTI, Seoul, Korea) as the counter electrode. In addition, full cells for evaluating life characteristics were assembled using LiNi_0.6_Mn_0.2_Co_0.2_O_2_ (NMC622) as the counter electrode. The cathode used in the full cells was a mixture of 94 wt.% NCM622 (Umicore), 3 wt.% conductive agent (Ketjenblack EC600JD, Lion Corporation, Tokyo, Japan), and 3 wt.% binder (PVDF, SOLEF). It was fabricated with an area capacity of 3.0 mAh/cm^2^ and anode to cathode electrode capacity balancing (N/P ratio) of 1.1:1. A separator (polypropylene, 3501, Celgard, Tokyo, Japan) with a diameter of 17 mm and thickness of 24 μm was placed between the working electrode and the counter electrode of each cell. The electrolytes of the half-cells were filled with 1.0 M LiPF_6_ in EC/DEC/FEC = 5/70/25 (v%), and the electrolytes of the full cells were filled with 1.0 M LiPF_6_ in EC/DEC/FEC = 25/70/5 (v%).

### 2.5. Electrochemical Investigations in Cells

#### 2.5.1. Half-Cell Setups

Electrochemical evaluation of the lithiation/de-lithiation cycle of the cells was conducted using a TOSCAT-3100 battery tester (TOYO SYSTEM, Fukushima, Japan). To evaluate the half-cells, lithiation/de-lithiation was performed at 0.1 C (C-rate). The lithiation (CC) cutoff voltage was set to 0.01 V, and the voltage was maintained until it reached 0.01 C (CV). The de-lithiation (CC) cutoff voltage was set to 1.5 V.

#### 2.5.2. Full-Cell Setups

The voltage was set to a range of 2.7–4.2 V during the full-cell evaluation. The C-rate for the formation cycle step consisted of 0.1 C for 1 cycle (lithiation and de-lithiation) and 0.2 C for 2 and 3 cycles. The lithiation cutoff was set to CC 2.7 V and CV 0.05 C, and the de-lithiation cutoff was set to CC 4.2 V. During long-term life evaluation of the cells after the formation cycle, the cutoff was set to 0.5 C for lithiation and 1 C for de-lithiation. The cutoff condition was set as the same as in the formation step. For full cells, 1 C was calculated to be the same as the specific current of 172 mA/g of the active anode material NMC622. In addition, to examine the rate performance characteristics of the cells, the cells were evaluated at various C-rates (Low–High–Low) under the conditions listed in Table 1.

## 3. Results and Discussion

### 3.1. Analysis of XRD Pattern

**Figure 2:**
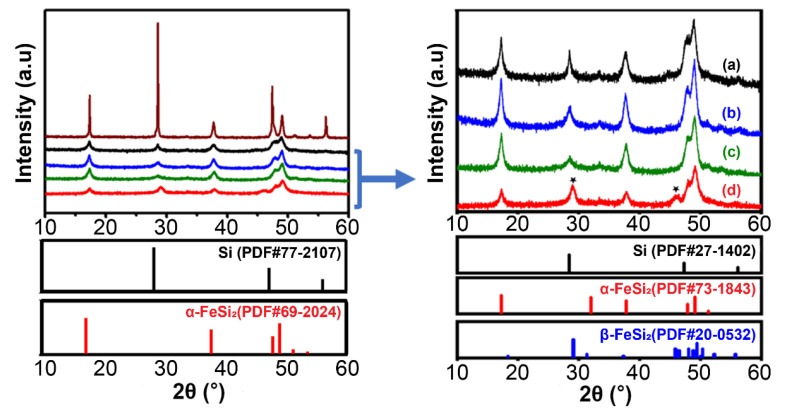
XRD patterns of Fe–Si alloy before and after milling.

The phase change according to the mechanical alloy milling time (2–24 h) of the Fe–Si alloy powders at an atomic ratio of 15:85 (weight ratio 25:75) was determined through the XRD pattern in Figure 2 and the XRD Rietveld analysis in Table 2.

The XRD analysis of the Fe–Si alloy powder before mechanical milling showed a pattern corresponding to Si (PDF#77-2107) and a sharp peak corresponding to α-FeSi_2_ (PDF#69-2024). However, after a short milling time of 2 h, the phase changed to Si (PDF#27-1402) and α-FeSi_2_ (PDF#73-1843) patterns and the intensity of the Si peak decreased significantly, indicating that sufficient milling energy had been applied. 

Additionally, as the milling time increased from 2 to 12 h Figure 2 (right, lines a–c), the active Si peak (2θ = 28.5) intensity tended to decrease and broaden. The crystal size continued to decrease to the critical value as the time of the mechanical alloying process increased. The additional milling energy supply causes the deformation of the crystals of the Si element and consequently to amorphization [26]. As shown in Table 2, even when the milling time increased, the content ratio of Si and the inactive iron silicide, a matrix, remained the same at approximately 25:75, as in the Rietveld analysis of XRD for each milling time. This indicated that the alloying process was completed in a short time (2 h). In the 24 h milled alloy powders shown in Figure 2 (right, line d), a new β-FeSi_2_ pattern (PDF #20-0532) was created, and the peak intensity of the α-FeSi_2_ phase decreased. This implied that some α-FeSi_2_ phases had been converted into β-FeSi_2_ phases.

According to the previous studies, FeSi_2_ exists in a metallic high-temperature α-FeSi_2_ phase and a semiconducting low-temperature β-FeSi_2_ phase. The α-FeSi_2_ phase has an Fe-deficient tetragonal structure, whereas the β-FeSi_2_ phase has an orthorhombic structure. As reported in the literature, the α-FeSi_2_ and β-FeSi_2_ phases of FeSi_2_ are formed during the ball milling of Si and Fe, respectively, and are converted to each other as soon as they are formed. However, the main cause behind the formation of one of the two phases under different milling conditions (e.g., milling time, heat generated during milling, and Si–Fe ratio) is unclear [34,35,36,37,38].

In this study, the conversion from the α-FeSi_2_ phase to the β-FeSi_2_ phase was examined, and only the milling time was increased while the other milling conditions remained the same. An active anode material with a stable structure was fabricated by forming nanocrystalline Si and a matrix of the α-FeSi_2_ phase surrounding the nanocrystalline Si through a mechanical alloy process. Over-milling converted some α-FeSi_2_ phases into β-FeSi_2_ phases.

### 3.2. Cross-Section Analysis

**Figure 3:**
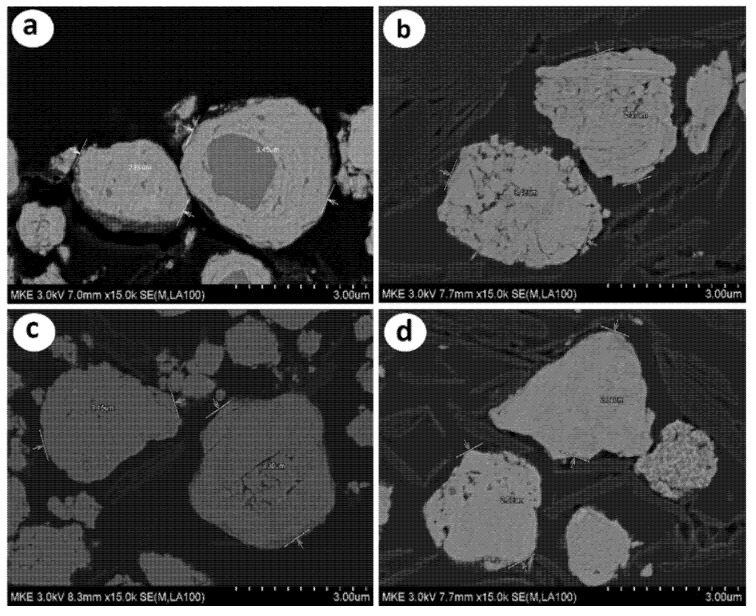
Cross-section images of Fe–Si alloy powders according to milling time: (**a**) 2 h milling, (**b**) 6 h milling, (**c**) 12 h milling, and (**d**) 24 h milling.

Figure 3 shows the SEM images of the cross-sections of the alloy powders with different milling times, and the effects of the milling process of the Fe–Si alloy powders can be clearly identified. In the XRD pattern of the 2 h milled alloy powder with a relatively low milling energy, there was no phase peak of Si and Fe elements that existed independently. However, in the SEM cross-sectional image in Figure 3a, some metal segregates that did not form the α-FeSi_2_ alloy could be seen. As mentioned above, it was estimated that the 2 h milled alloy powders were not in a perfect alloy state because there were segregates, although sufficient milling energy had been applied as the phase changed and the intensity significantly decreased [25]. Segregates were not observed on the cross-sections of the 6 h milled powders in Figure 3b. However, pores and cracks were present inside the particles because they repeatedly underwent pulverization and agglomeration during the milling process. Relatively dense particles were observed on the cross-sections of the 12 and 24 h milled powders in Figure 3c,d, and a stable form of alloy powders with no metal segregates could be seen [26].

**Figure 4:**
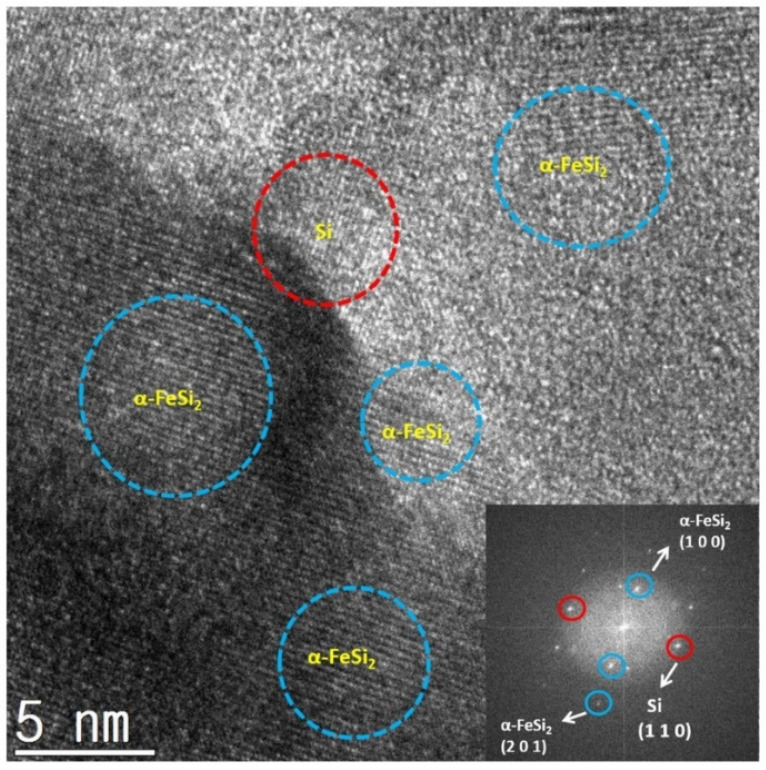
TEM images and grid structure analysis of 12 h milled Fe–Si alloy powders.

Figure 4 shows the TEM analysis result for the 12 h milled powders, confirming the stable structure of Si surrounded by α-FeSi_2_ phases. Thus, buffering the expansion of Si due to the insertion of Li ions during lithiation may be expected. Next, the effect of the iron silicide matrix (α-FeSi_2_, β-FeSi_2_) phase conversion on the active anode material was investigated by evaluating its electrical conductivity and electrochemical properties. 

### 3.3. Resistance Analysis

**Figure 5:**
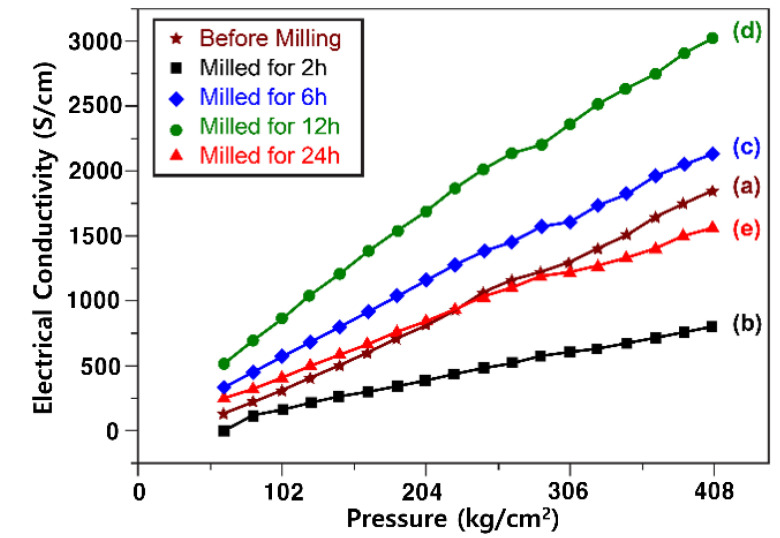
Electrical conductivity graph of Fe–Si alloy powders according to the compression of powders. (**a**) Before milling and after milling for (**b**) 2, (**c**) 6, (**d**) 12, and (**e**) 24 h.

The changes in the electrical conductivity of the iron silicide matrix (α-FeSi_2_, β-FeSi_2_) owing to the alloy state and phase conversion were observed by measuring the resistance of the alloy powder according to the milling time, as shown in Figure 5. To equalize the interfacial contact area between the powders when measuring the powder resistance, a pressure of up to 408 kg/cm^2^ was applied during the measurement. The electrical conductivity of the powders based on a compression of 408 kg/cm^2^ decreased significantly, from 1843 S/cm before milling, to 798 S/cm after milling for 2 h. This appeared to be due to the fact that Fe (96.1 n Ω·m), which had a high conductivity, was alloyed to the FeSi_2_ phase, and there were some segregates. The electrical conductivity increased significantly to 2121 and 3018 S/cm after milling for 6 and 12 h, respectively. This was attributed to uniform alloying and the formation of a solid network of FeSi_2_ phases. However, when the milling time was increased to 24 h, the electrical conductivity significantly decreased to 1526 S/cm, which is a level below the electrical conductivity obtained before milling. This result can be attributed to the semiconducting characteristics of the β-FeSi_2_ phase caused by over-milling [39,40,41,42].

### 3.4. Electrochemical Performance

**Figure 6:**
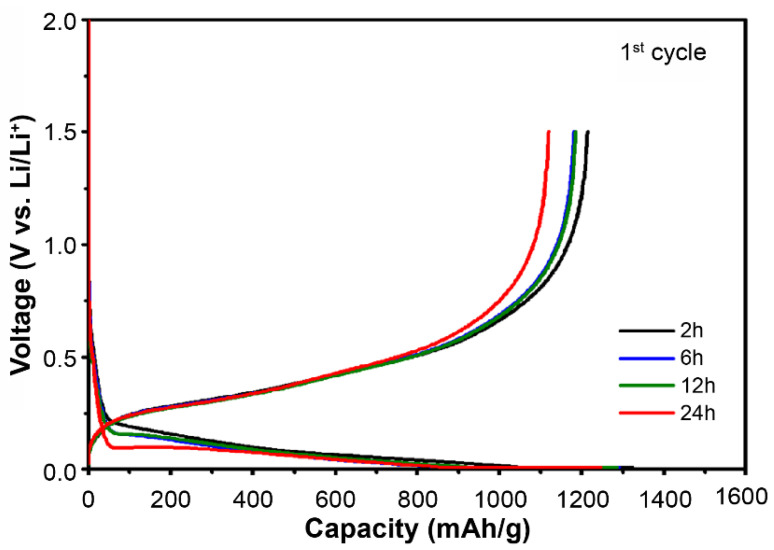
Potential profiles of half-coin cells, showing the first cycle for Fe–Si alloy powders milled at different times of 2, 6, 12, and 24 h during lithiation/de-lithiation at a specific current of 100 mAh/g to determine the specific capacity of the materials.

The initial capacity and efficiency characteristics according to the milling time of the half-coin cells that were fabricated by adding a PAA binder, a conductive material, and 80 wt.% of Si, and that underwent lithiation/de-lithiation at a rate of 0.1 C are shown in Figure 6. Table 3 lists the lithiation and de-lithiation capacities and initial efficiencies (de-lithiation/lithiation capacity × 100) of each powder. The de-lithiation capacity of the 2 h milled Fe–Si alloy powder was 1215 mAh/g, and that of the 6 and 12 h milled Fe–Si alloy powders was approximately 1185 mAh/g, which slightly decreased. As shown in the cross-sectional image of the powders, the ratio of active Si reacting with Li decreased slightly because incompletely alloyed active Si was alloyed to iron silicide throughout a milling time of 2 h. Finally, the 24 h milled Fe–Si alloy powder showed a significant decrease in both lithiation and de-lithiation capacities. This can be interpreted to indicate that the converted β-FeSi_2_ phase matrix of semiconductor properties increased the relative resistance to the reaction between Si and Li [37,43].

The initial capacity and efficiency of the cells are very important in determining the characteristics of the anode material [44,45]. Through this evaluation, it can be seen that the β-FeSi_2_ phase, which was formed and converted during over-milling (more than 12 h) in the mechanical alloying process, was the cause of the specific deterioration.

**Figure 7:**
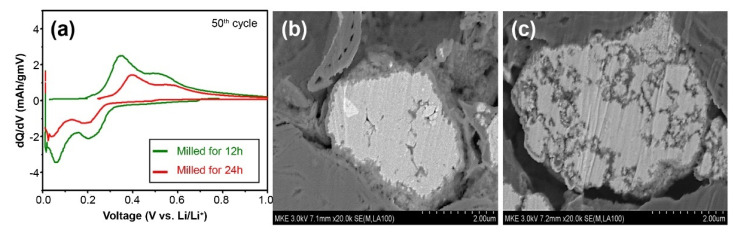
(**a**) Differential capacity plots (DCPs) at 50 cycles of charge/discharge of the 12 and 24 h milled powders. (**b**) SEM image after 50 cycles of charge/discharge of 12 h milled powders. (**c**) SEM image after 50 cycles of charge/discharge of 24 h milled powders.

Figure 7 shows the dQ/dV graph of 50 cycles of lithiation and de-lithiation in the half-coin cells assembled using the same method as described above for the 12 and 24 h milled mixed powders and the SEM images of particle cross-sections after 50 cycles. For the evaluation condition, one cycle of lithiation/de-lithiation was performed (formation) at a rate of 0.1 C, and then 50 cycles were carried out at a rate of 0.5/1 C. To examine the effect of the milling time on the converted β-FeSi_2_ matrix phase, changes in the electrochemical behaviors and microstructures inside the particles during lithiation/de-lithiation after 50 cycles were analyzed. The de-lithiation peak was around 0.2–0.6 V, and it could be seen that the peak intensity of the cell fabricated by the 24 h milled mixed powder of the β-FeSi_2_ matrix structure was significantly low. Furthermore, regarding the particle cross-section after the cycle, a side reaction material (SEI layer) with the electrolyte was thinly formed only on the surface of the 12 h milled powder, whereas a large amount of this material was formed inside as well as on the surface of the particles of the 24 h milled powders. This was considered to be due to the fact that the β-FeSi_2_ matrix of semiconductor properties not only had low electrical conductivity, but was also vulnerable to volume changes due to the expansion/contraction of particles during lithiation/de-lithiation, thus causing internal cracks. The reaction with the electrolyte may have caused continuous irreversible reactions and depletion of the electrolyte at the newly formed active interface through such cracks [46,47,48].

In this study, an electrolyte was injected and electrochemically evaluated. However, it is believed that even when an electrolyte injection is administered to commercially available pouches and cylindrical or prismatic full cells, if the injection is difficult owing to the design, it will lead to the deterioration of the long-term life characteristics of the cells.

**Figure 8:**
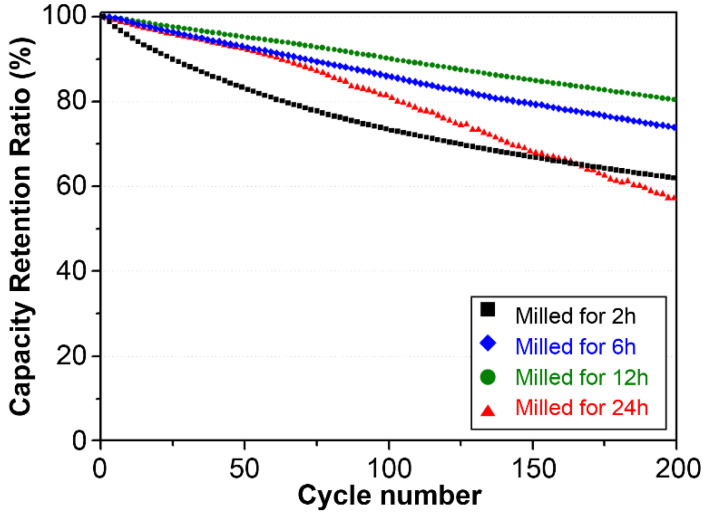
Cyclic performance of Fe–Si alloy powders according to milling time.

Next, full coin cells were assembled by adding SBR, CMC, conductive material, 15% Fe–Si alloy, and 81% powdered graphite to evaluate the life and rate performance characteristics of these cells. Then, in the formation step, cycle evaluation was performed using 1 cycle of 0.1 C lithiation and de-lithiation, 2 cycles of 0.2 C lithiation and de-lithiation, 0.5 C lithiation, and 1.0 C de-lithiation. As shown in Figure 8, the 2 h milled Fe–Si alloy powder displayed a large, irreversible capacity from the beginning. This appeared to be because a stable structure could not be maintained owing to the large number of segregates and large Si crystallite size compared to the Fe–Si alloy powders milled for 6 to 24 h. The life characteristics of the 6 and 12 h milled Fe–Si alloy powders were greatly improved, and the 12 h milled Fe–Si alloy powder, which had a relatively small crystallite size and dense microstructure, showed the highest retention rate. This result was attributed to the maintenance of structural stability by the minimized expansion caused by the nanocrystalline Si and the improved internal strength of the particles.

Meanwhile, the life retention rate of the 24 h milled alloy powder converted to the β-FeSi_2_ matrix phase showed a sharp decline. This could be interpreted based on the SEM analysis result for the cross-section of the half-coin cell (PAA binder) fabricated with a high ratio (80 wt.%) of Fe–Si alloy powders described above (Figure 7). In the 24 h milled alloy powder, a continuous irreversible capacity was generated as a new active interface emerged after the matrix structure collapsed owing to repetitive lithiation/de-lithiation because the ion conduction path was disturbed, and the particle strength was relatively low owing to the semiconducting matrix. The above discussion confirmed that the formation of a solid matrix of α-FeSi_2_ was crucial for the formation of a stable structure that buffered the expansion of Si.

**Figure 9:**
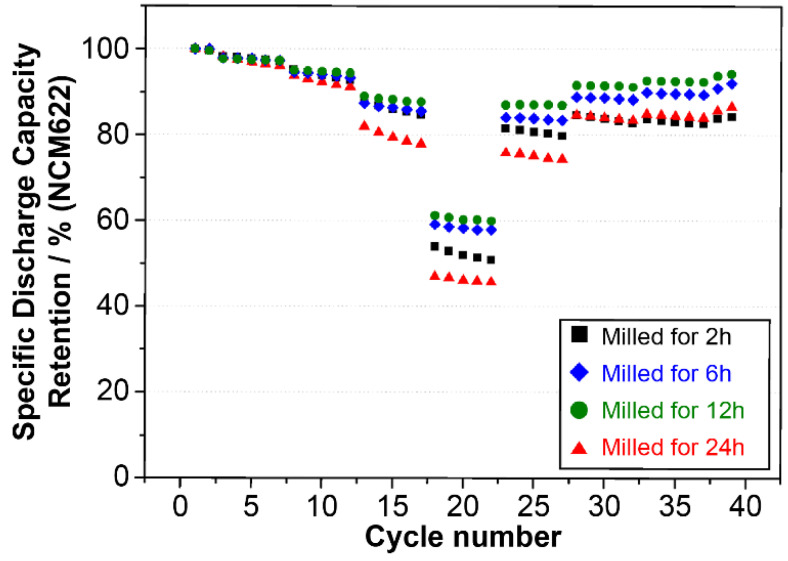
Constant current charge/discharge cycling experiments for full coin cells at different C-rates.

The evaluation results of the rate performance characteristics of the Si-Fe alloy powders are shown in Figure 9. The Fe–Si alloy powder milled for 12 h at 3 C (high C-rate) showed the most stable properties, but the alloy powder milled for 24 h with the FeSi_2_-b matrix structure showed relatively poor properties. After a high C-rate up to 3 C, the 24 h milling sample in the recovery evaluation at 0.1 C was 86%. This is interpreted as a structural collapse caused by local expansion at 3 C (high C-rate) due to low structural stability.

## 4. Conclusions

In this study, we manufactured nanocrystalline Fe–Si alloy powders as active anode materials for Li secondary batteries through a mechanical alloying process using Si and Fe alloy powders. The Fe–Si alloy powders underwent nano-crystallization of Si and a phase change in the FeSi_2_ matrix according to the mechanical milling time. The resulting changes in the electrochemical properties of the powders were investigated. The XRD patterns and Rietveld analysis of the nano-crystallization of Si and the phase change of the matrix confirmed that as the milling time increased from 2 to 12 h, the Si crystallite size decreased, but the α-FeSi_2_ matrix remained the same. However, when the milling time was increased to 24 h or more, the alloying α-FeSi_2_ matrix was converted to a semiconducting low-temperature β-FeSi_2_ phase. The effects of the nano-crystallization of Si and the conversion of the matrix on the alloy powders were investigated. First, as the degree of nano-crystallization of Si increased, the lithiation/de-lithiation capacities became similar, but the initial efficiency tended to increase. Furthermore, it was found that the life retention rate improved significantly even after repeated lithiation/de-lithiation cycles. In contrast, the semiconducting β-FeSi_2_ phase matrix generated in the 24 h milled alloy powders, for which an excessive milling time was applied, deteriorated the chemical reaction of Si and Li due to low electrical conductivity, thus decreasing the capacity of the cells. Moreover, it caused a rapid reduction in the long-term life characteristics of the cells because it broke down the stable structure of the α-FeSi_2_ phase and could not buffer the Si expansion problem. These results provided insight into improving battery performance through the commercialization of Si materials. 

While previous investigations and studies focused on the production of Fe–Si alloy powders with an optimal and stable phase using the mechanical alloying process, this study detailed a new phase of over-milling (increasing milling time) during the mechanical alloying process. The electrochemical degradation characteristics of the alloy powders according to the formation and conversion of their phases were investigated.

Based on these results, it was found that the formation and conversion of the semiconducting, low-temperature β-FeSi_2_ phase should be prevented to manufacture an excellent anode material for an Fe–Si alloy by utilizing the mechanical alloying process. Investigations and studies on the effects of the stable matrix phases of Si and iron silicide on the electrochemical properties of Fe–Si alloy powders are needed to further improve the long-term cycle performance of the cells.

## Figures and Tables

**Table 1:** C-rate condition setting for the evaluation of rate performance characteristics.

Evaluation Conditions		Division
C-Rate	Number of Lithiation/De-Lithiation Cycles	
0.1 C	1 cycle	Acceleration
0.2 C	1 cycle	
0.5 C	5 cycles	
1.0 C	5 cycles	
2.0 C	5 cycles	
3.0 C	5 cycles	
2.0 C	5 cycles	Recovery
1.0 C	5 cycles	
0.5 C	5 cycles	
0.2 C	1 cycle	
0.1 C	1 cycle	

**Table 2:** Weight ratios of FeSi_2_ and Si and Si crystallite size by milling time determined through Rietveld analysis.

Milling Time (h)	Formula (wt.%)		Si Crystallite Size (nm)
	FeSi_2_	Si	
2	74.1	25.9	5.8
6	74.0	26.0	3.86
12	74.0	26.0	2.47
24	74.6	25.4	2.27

**Table 3:** Table of half-coin cells, showing the first cycle for Fe–Si alloy powders milled at different times of 2, 6, 12, and 24 h during lithiation/de-lithiation at a specific current of 100 mAh/g to determine the specific capacity of the materials.

	2 h	6 h	12 h	24 h
Charge capacity (mAh/g)	1323	1291	1286	1220
Discharge capacity (mAh/g)	1215	1181	1186	1120
Initial Coulombic Efficiency (%)	91.8	91.5	92.2	91.8

## Data Availability

Data sharing is not applicable for this paper.
